# Human biliary atresia extrahepatic cholangiocyte organoids express increased ER and oxidative stress, altered drug metabolism and cell polarity changes

**DOI:** 10.3389/fbioe.2026.1777423

**Published:** 2026-05-07

**Authors:** Adi Har-Zahav, Yara Hamoudi, Keren Danan, Ana Tobar, Michal Bespalchik, Michael Gurevich, Raanan Shamir, Irit Gat-Viks, Orith Waisbourd-Zinman

**Affiliations:** 1 Gastroenterology, Nutrition and Liver Diseases, Schneider Children’s Medical Center, Petach-Tiqva, Israel; 2 Felsenstein Medical Research Center, Gray Faculty of Medical and Health Sciences, Tel-Aviv University, Tel-Aviv, Israel; 3 The Shmunis School of Biomedicine and Cancer Research, George S. Wise Faculty of Life Sciences, Tel Aviv University, Tel Aviv, Israel; 4 Department of Pathology, Beilinson Medical Center, Petach-Tiqva, Israel; 5 Pediatric Liver Transplant Unit, Schneider Children’s Medical Center, Petach-Tiqva, Israel; 6 Children’s Hospital of Philadelphia, University of Pennsylvania, Philadelphia, PA, United States

**Keywords:** biliary atresia, cell polarity, cholangiocyte, drug metabolism, er stress, extrahepatic cholangiocyte organoids

## Abstract

Biliary atresia (BA), the leading cause of pediatric liver transplantation, is characterized by neonatal jaundice and progressive extrahepatic bile duct obstruction, yet its pathogenesis remains elusive. To uncover extrahepatic cholangiocyte injury, we generated patient-derived extrahepatic cholangiocyte organoids (EHCOs) from bile duct remnants of BA and controls. We performed discovery bulk RNA-seq followed by targeted molecular and functional validation. Transcriptomic profiling and pathway/perturbation analyses revealed dysregulation of ER stress pathways, xenobiotics/drug metabolism, and genes regulating cell adhesion and polarity. BA EHCOs exhibited altered expression of E-cadherin, RhoU, SOX17, and CFTR. ER stress markers PERK, BiP, and ATF4 were elevated, and TEM demonstrated abnormal ER morphology. Conversely, CHOP, ERO1A, WFS1, and SOD3 were downregulated, suggesting impaired ER stress resolution. Functionally, BA EHCOs were more susceptible to toxic injury, and CYP450 inhibition attenuated ER stress genes expression. Immunostaining of BA liver hilum biopsies confirmed elevated PERK and BiP. Together, these data identify unknown arms of ER stress and epithelial dysfunction as disease-relevant injury process of the extrahepatic biliary tree and potentially implicate xenobiotic metabolism as a modifiable driver.

## Graphical Abstract

**Figure:**
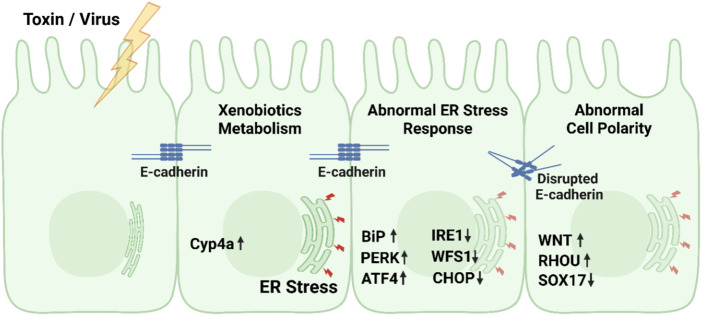

## Introduction

Biliary atresia (BA) is a poorly understood cholangiopathy, characterized by progressive extrahepatic bile duct obstruction in newborns. It is the leading cause of pediatric liver transplantation worldwide. Despite numerous years of research, the etiology and pathogenesis of BA remain elusive. The primary treatment is the Kasai portoenterostomy (KPE), a surgical attempt to restore bile flow from the liver to the intestine; however, outcomes remain unsatisfactory for many patients (Wehrman et al., 2019). A deeper understanding of the pathogenesis of BA, specifically understanding molecular mechanisms of extrahepatic bile duct injury in BA is critical for developing future therapeutic strategies (Bezerra et al., 2018).

Clinically, the phenotype of BA at the time of presentation is more pronounced in the extrahepatic biliary tree compared to its intrahepatic counterpart. Even in cases where bile flow is successfully restored following KPE, patients often continue to experience progressive biliary fibrosis. BA is thus considered a pan-cholangiopathy, affecting both the intrahepatic and extrahepatic biliary systems. It is worth noting that the intra- and extrahepatic bile ducts originate from different embryonic sources. The extrahepatic ducts develop alongside the ventral pancreatic ductal system, later connecting with the intrahepatic ducts that originate from the hepatic bud (Raynaud et al., 2011). This distinction in embryological origin suggests that the molecular processes in the extrahepatic biliary tree during BA may differ from those in the intrahepatic bile ducts, necessitating further investigation, which is the focus of this study.

In recent years, transcriptomics has provided significant insights into the molecular landscape of BA. High-throughput RNA sequencing (RNA-seq) has identified differentially expressed genes, pathways, and potential molecular mechanisms underlying BA pathogenesis. Previous studies have highlighted immune-mediated damage (Wang et al., 2020), the interplay between fibrosis and the immune system, (Ye et al., 2022) and abnormalities in ciliary function and epithelial cell development (Glessner et al., 2023). While these insights have expanded our understanding of BA, they primarily reflect secondary consequences of bile duct obstruction rather than the initial pathogenic events. Given that BA manifests first in the extrahepatic biliary tree, where bile duct injury is most pronounced, it is essential to specifically investigate this compartment.

In this study, we used extrahepatic cholangiocyte organoids (EHCOs) derived from BA patients undergoing KPE and compared them with normal cholangiocyte organoids from patients undergoing liver transplantation for metabolic conditions. Through transcriptomic profiling, advanced analysis, and functional validation, we aimed to investigate the mechanisms of injury in the extrahepatic biliary tree in BA. To our knowledge, this is the first study to compare the transcriptome of extrahepatic cholangiocyte organoids from BA patients with those from non-BA controls.

## Materials and methods

### Organoids isolation and growth

#### Primary biliary tissue

Primary extrahepatic biliary tissue was obtained from patients undergoing Kasai procedure, or liver transplantation, under an approved Institutional Review Board protocol (RMC 19-0072) (Supplementary Table S1). The common bile duct was excised and placed immediately in UW solution for EHCOs generation.

#### EHCOs generation and propagation

EHCOs were generated and propagated according to Tysoe et al. (2019) Briefly, the common bile ducts were dissected and washed and the luminal epithelium was scraped using a surgical blade and covered with William’s E media (Gibco). The cells were centrifuged at 500 g for 4 min, washed and resuspended in a mixture of 66% Matrigel (Corning, 356,237) and 33% supplemented William’s E medium (listed under Supplementary Table S6 in Supplemented Materials). 50µL of the cell suspension was plated in a 24-well plate so that a small dome of Matrigel was formed in the center of each well. Plates were incubated at 37 °C for 2 min, then placed upside down and incubated for an additional 30 min until the Matrigel was solidified. Subsequently, 1 mL of William’s E medium with supplements was added to each well. 10 μM Y-27632 (Corning, 354,352) was added for 48 h post seeding. The culture medium was replaced three times a week. Confluent wells were split at a 1:4 ratio, and EHCOs were cryopreserved using NutriFreez D10 Medium (Sartorius, 05-713-1A) at −80^0^c, then transferred into liquid nitrogen for long-term preservation. All experiments were performed using EHCOs cultures that were previously cryopreserved and thawed in order to maintain consistent conditions. Organoid morphology was assessed using brightfield microscopy, and images were taken using ZEISS Primovert light microscope equipped with an Axiocam 105 color camera, using ×4 and ×20 objectives. HCOs were characterized using immunofluorescence staining for CK19 and albumin (ALB), as detailed below.

### Bulk RNAseq

#### RNA extraction and RNA bulk-sequencing

RNA was extracted from five BA and three non-BA controls EHCOs at passage 2, upon confluency (7–10 days after passaging),using the EZ-10 DNAaway RNA Miniprep Kit (BIO BASIC #BS88136) according to the manufacturer’s protocol. RNA integrity was analyzed using TapeStation (Agilent Technologies), and samples (RIN>8) were sent to the Crown Genomics Institute of the Nancy and Stephen Grand Israel National Center for Personalized Medicine (G-INCPM) and Weizmann Institute of Science for library preparation and sequencing. The samples were sequenced using Illumina NextSeq High Output (-75 cycles).

#### RNA-seq data of BA patients

The dataset consists of bulk RNA-seq profiles from EHCOs originating from patients with BA undergoing Kasai portoenterostomy and normal ducts from patients undergoing liver transplantation for metabolic conditions. For all samples, we used FASTQ files of the forward DNA strand (length 67bp). Quality control was performed using FastQC. As evidence for the quality of data, we observed the following metrics: on average, each sample contains 30.7 M reads, with no reads flagged as poor quality, and the average GC content across samples was 51%. Alignment of reads to the reference genome (hg38. refGene) was conducted using the STAR aligner, resulting in a reads-per-gene count matrix. Both the raw sequencing data and the count matrix were deposited in GEO (accession number GSE276230). The average mapping ratio was 83.54%, with a total of 28,271 genes detected. Gene filtration and normalization were performed using the DESeq2 R package (Love et al., 2014), with default filtering settings and a minimum read count threshold of 10 reads per gene. After filtration, 16,896 genes were retained for subsequent analysis.

### Analysis of differential expression and activation in BA

We applied two complementary analyses: “differential expression” and “differential activation”. In the differential-expression analysis, we directly compared gene-expression levels between BA and healthy controls to identify genes that are upregulated or downregulated in BA. In the differential-activation analysis, we integrated these BA-associated expression changes with an external genome-wide perturbation dataset that captures the transcriptional consequences of perturbing individual genes, allowing us to infer which regulators are predicted to be activated or repressed based on the transcriptional changes in BA. In this way, ‘differential expression’ describes the observed transcriptional changes, whereas ‘differential activation’ points to the changes in upstream factors that may underlie the observed transcriptional changes.

1. Differential expression. Differential expression (DE) analysis of BA *versus* controls was performed through DeSeq2 in R (Supplementary Table S2). Because all samples were processed together throughout the entire experimental pipeline, we did not include a batch covariate in this DE analysis. For the log2FC value, positive/negative sign indicates upregulation or downregulation in BA compared to non-BA samples. For each pathway (from KEGG and the GO repositories), we calculated the bias of the log2FC scores for genes within the pathway compared with the remaining genes. The bias is quantified using the Wilcoxon rank-sum test (FDR-adjusted *q* values). The ‘differentially expressed pathways’ are pathways with *q*-value <0.005. Enriched pathways are further classified as either upregulated or downregulated pathways according to the direction of bias in BA–namely, ‘upregulated/downregulated pathways’ in BA are those that are enriched with genes that have high/low log2FC scores (Supplementary Table S3).
2. Differential activation. In the following, we first describe the differential-activation analysis, which takes as input the BA gene sets as well as “perturbation effect score” profiles (2.1). The ‘perturbation effect score’ is generated based on analysis of an external perturbation dataset. We provide details on the preprocessing of this external perturbation dataset (2.2), and on how this external dataset was used to generate the ‘perturbation effect score’ profiles (2.3). In practice, we first preprocessed the external dataset (2.2), then derived the perturbation-effect score profiles (2.3), and finally used the calculated profiles as input for the differential-activation analysis (2.1).
  2.1 Calculation of differential activation scores. The analysis takes as input: (i) A BA gene sets: either a BA-upregulation gene set, including the 100 genes with highest log_2_FC scores (denoted BA-Up, log_2_FC > 4.4), or the BA-downregulation gene set, including the 100 genes with lowest log_2_2FC scores (denoted BA-Down, log_2_FC < −2.291) (Supplementary Table S2). (ii) A ‘perturbation effect score’ for each perturbed gene *r* (called ‘factor’) on the expression of each gene *g*. Each ‘perturbation effect score profile’ *r* consists of the effect of perturbation in factor *r* on each of 8,748 transcribed genes (see Section 2.3). Of note, only 18 of the 100 BA-Up genes and only 10 of the 100 BA-Down genes were expressed in the external perturbation dataset and were therefore used in the subsequent calculation. Given an input BA gene set (BA-Up or BA-Down sets) and a perturbation effect score profile of a given factor, the ‘effect of perturbation on a BA gene set’ is defined as the bias of the perturbation effect scores of the perturbed factor on genes within the BA gene set compared to the remaining genes (a Wilcoxon rank-sum test *p* value) (Supplementary Table S4). Thus, a perturbation upregulates/downregulates the BA gene set when the genes in the BA gene set tend to high/low perturbation effect scores. We distinguish four types of factors with a significant perturbation effect (Supplementary Table S4): Factors whose perturbation (1) downregulates the BA-Down’s genes (25 factors, effect on BA-Down *p* < 0.11); (2) downregulates the BA-Up’s genes (48 factors, on BA-Up p < 0.01); (3) upregulates the BA-Down’s genes (25 factors, effect on BA-Down p < 0.07); and (4) upregulates the BA-Up’s genes (2 factors, effect on BA-Up p < 0.08). For the factors within each of these categories, we performed hyper-geometric test using each of the pathways of the REACTOME collections (Supplementary Table S5). Pathways that are enriched (q < 0.05) are referred to as ‘differentially activated pathways’.
  2.2 Details on the pre-processing of the external perturbation dataset. We used perturb-seq data, which targets genes with CRISPR interference (CRISPRi) with subsequent transcription phenotyping at single cell resolution (Replogle et al., 2022). The experiment was performed in the retinal pigment epithelial (RPE1) cell line. It is the largest single-cell CRISPRi Perturb-seq resources available, with systematic transcriptional consequences of thousands of perturbations in a polarized epithelial context. We treated the Perturb-seq resource as a cross-epithelial reference because many core epithelial programs behave similarly across tissues. We identified the top up- and downregulated genes in BA EHCOs (detailed in 2.1) and assessed their concordance with gene-expression changes induced by specific CRISPRi knockdowns. We used as input the processed data after various filtration and pre-processing steps as reported in Replogle et al. (2022). The input data, which is used as input in our study, consists of a z-score for each gene in each single cell. The z-scores were obtained by normalization to control cells. The input data include 2,393 perturbations, each of which was measured in 2–3,461 single cells; each single cell is represented by a vector of z scores across 8,748 transcribed genes. In addition, the input data includes non-targeting control cells.
  2.3 The calculation of ‘perturbation effect score’ profiles. For each genetic perturbation in gene *r*, we applied the following two steps. First, we calculated the effect of the perturbation in gene *r* on each transcribed gene *g* by comparing the z scores of gene *g* in all single cells in which *r* is perturbed against all non-targeting (control) single cells (p-value of Wilcoxon rank-sum test, q-value after FDR correction for multiple genes). The ‘perturbation effect score’ of a perturbation *r* on a gene *g* is defined as the signed log10 of this *q*-value, with positive/negative perturbation effect scores for increase/decrease in the median values of gene *g* in *r*-perturbed cells compared to non-targeting cells. Each perturbation *r* is represented with a ‘perturbation effect score profile’, consisting of a 8748-length list of the perturbation effect scores for perturbation *r* across all 8,748 transcribed genes. A total of 2,393 perturbation profiles were calculated, a profile for each perturbed gene. These profiles were used as input in the analysis of differential activation in Section 2.1.

### EHCOs immunofluorescent (IF) stains and microscopy

EHCOs were plated on 15 well and 18 well µ-slide with glass bottom (ibidi #81817, #81507) in a 10 µL mixture of 2:1 Matrigel and E+ supplemented media, covered after solidifying with additional 50 µL supplemented media. Media was replaced every 48 h for 7–10 days. EHCOs were fixed using 4% PFA for 20 min at 4^0^c, followed by three washes with 1xPBS. EHCOs were incubated with sera containing permeabilization solution (10% FCS, 1% BSA, 2% goat serum, 0.2% TTX) for 1 h at room temperature, then antibodies (Supplementary Table S8) were added and incubated over-night at 4^0^c, followed by three washes with permeabilization solution (45 min each, at room temperature). Secondary antibodies were added along with phalloidin and incubated overnight at 4^0^c. The next day, the slides were washed 3 times with PBSx1, stained with DAPI for 10 min at room temperature, washed again, and covered with PBSx1. For EHCO characterization, directly conjugated antibodies were used. Antibodies were incubated at room temperature for 2 h, followed by three washes with 1× PBS and nuclear staining with DAPI. FFPE liver sections were used as positive controls and stained under the same conditions. Images were generated using a Leica SP8 confocal microscope, at ×20 and ×40 magnification, and STELLARIS confocal microscope at ×25 magnification, images were analyzed using ImageJ software. Analyses were performed on passage-matched, morphologically intact, growth-competent EHCOs from BA and control donors.

#### Rhodamine efflux assay

Control and BA EHCOs were seeded on a glass-bottom 15-well µ-Slide (ibidi, 81,507). In order to assess monolayer permeability, EHCOs were treated with 20 μM Rhodamine 123 (Merck, 83,702) for 30 min, followed by three washes with supplemented William’s E media. EHCOs were then imaged using a STELLARIS confocal microscope equipped with an OKOLAB Cage incubator system at 37 °C in a 5% CO_2_ atmosphere. Images were taken using an L-25x/0.95 W VISIR 0.17 objective every 5 min for 4 h.

#### Real time quantitative PCR

EHCOs were harvested using Cell Recovery Solution (Corning) and RNA was extracted using the RNeasy Mini Kit (QIAGEN). cDNA was prepared using the qScript TM cDNA Synthesis Kit (Quanta Bio). Quantitative PCR was performed using StepOne Plus and SYBR Green reagent (Applied Biosystems). The primers used are listed in Supplementary Table S7. Expression analysis was performed using the double ΔCt method.

#### ER immunofluorescence stain

EHCOs were harvested using Cell Recovery Solution (Corning), placed on ice for 30 min, then washed, and incubated in 1 mL TrypLE Express Enzyme (Gibco) for 5 min at 37^0^c. Following pipetting, another 1 mL supplemented William’s E media was added, and the cells were strained using a pre-washed 30 μm cell strainer (PluriSelect). 100,000 cells per well were seeded as monolayer on a Lab-Tek II eight well plate (Nunc) covered with 1 mg/mL rat tail collagen. Cells were fixed with 3.6% PFA and stained using the ER-ID green assay kit (ENZO #51025) according to the manufacturer’s protocol. Images were generated using a Nikon AX confocal microscope at ×60 magnification and processed using ImageJ software.

#### TEM microscopy

EHCOs were harvested and placed in Karnovski (Glutarhaldehide 70%, Formalin 37%, Cacodylate buffer0.2 M,H_2_O), and transferred to the Rabin medical center TEM pathology core facility to prepare for microscopic imaging. Images were taken using a JEM-1400Flash microscope.

#### HET0116 treatment

EHCOs were treated with 8ug/mL Cytochrome P450 inhibitor, HET-0016 (CAS 339068-25-6, Santa Cruz) for 6 h. EHCOs were harvested and RNA was extracted. ER stress-related genes expression was measured by RT-qPCR and compared to untreated EHCOs.

#### Biliatresone treatment

EHCOs cultures were treated with either five or 10 ug/mL biliatresone (HY-119412, MCE) for 72 h. An equivalent volume of DMSO was used as a vehicle control. The effect of biliatresone was assessed by evaluating EHCOs integrity or IF staining. To assess organoid integrity, brightfield images were acquired 72 h post-treatment using a ZEISS Primovert light microscope equipped with an Axiocam 105 color camera and a ×4 objective. EHCOs were classified as either intact or deformed and quantified using ImageJ software. For each condition, at least 35 EHCOs were analyzed per culture across three independent experiments.

For IF staining, EHCOs were fixed with 4% PFA and processed as described in the “IF Stains and Microscopy” section. This analysis was performed in five independent experiments.

#### Primary murine neonatal extrahepatic cholangiocytes culture

Primary neonatal (3 days old) extrahepatic cholangiocytes were extracted from blb/c mice and cultured in biliary epithelial cell (BEC) medium at 37 °C with 5% CO_2_ as previously described (Waisbourd-Zinman et al., 2016), Cells were left untreated or exposed to 1 μM thapsigargin (Sigma-Aldrich, T9033) or 5 μg/mL biliatresone for 1, 2, and 6 h, and eIF2α phosphorylation was assessed by Western blot as described below.

All mice used in experiments were under a strict standard of care and experimental planning, covered by licensed approval from the Tel Aviv University Institutional Review Board and the Israeli Ministry of Health (License number 2306-138-1). BALB/c mice were obtained from ENVIGO Israel. Both male and female mice were used for all analyses.

#### Staining of paraffin-embedded human slides

Paraffin-embedded slides from the liver hilum obtained from BA patients and non-BA controls were stained for PERK and BiP antibodies (Supplementary Table S8), as previously described (Fried et al., 2020). Images were captured using a Nikon AX Confocal microscope at ×40 magnification and analyzed using ImageJ.

#### Western blotting

For western blots, cells were lysed in 2× RIPA Buffer I (pH 7.4; Bio Basic, RB4475) supplemented with 40 μL/mL Roche cOmplete™ protease inhibitor (Sigma-Aldrich, 11,697,498,001) for 30 min on ice. Lysates were centrifuged at 14,000 rpm for 15 min, and the supernatant was collected for further analysis. Total protein concentration was measured using Bradford Reagent (Sigma-Aldrich, B6916) according to the manufacturer’s protocol, and 30–50 μg of protein per sample were separated by sodium dodecyl sulfate–polyacrylamide gel electrophoresis (Invitrogen, NW00102BOX), transferred to a 0.2 μm polyvinylidene difluoride membrane (Bio-Rad, 162–0176), blocked with skim milk, and incubated with primary antibodies against E-cadherin (1:500, Abcam, ab231303), Phospho-eIF2alpha (MBL, AT-6031), total eIF2alpha (Cell Signaling, L57A5) and glyceraldehyde 3-phosphate dehydrogenase (GAPDH; 1:5,000, Cell Signaling, 2,118) as a loading control. Signals were detected using fluorescence on a Li-Cor Odyssey system, and band densitometric analysis was performed using ImageJ software.

#### Statistical analysis

For the experiments other than the RNA-seq analysis, unless stated otherwise, the mean, standard error, and p values were calculated using GraphPad Prism 9. Statistical significance was calculated by unpaired Student’s t-test, and a p value < 0.05 was considered statistically significant. Normal distribution was assessed using Shapiro-Wilks normality test. For datasets that did not meet the assumptions of normality, nonparametric comparisons were performed using the Mann–Whitney test. Given the limited number of human samples for the verification analysis, statistical power is modest and results should be interpreted with caution.

## Results

### BA extrahepatic cholangiocyte organoids differ from normal controls in morphology and gene expression

**FIGURE 1:**
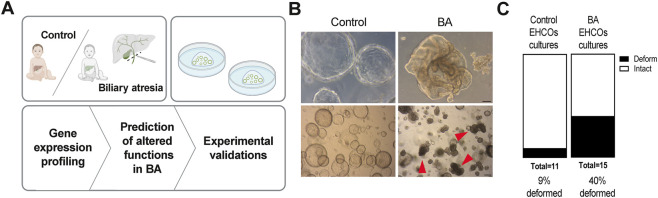
Study design and cholangiocyte organoids propagation **(A)** Study design: extrahepatic bile ducts were obtained from BA patient and non-BA controls and propagated as human cholangiocyte organoids (EHCOs). EHCOs underwent bulk RNA-seq and transcriptomic analysis **(B)** Representative images of intact control EHCOs, and deformed BA EHCOs. Red arrowheads indicate of obstructed EHCOs, upper panel scale bar = 200 um, lower panel scale bar = 100 um **(C)** Quantification of EHCOs morphology as open lumens or deformed shapes in BA EHCOs cultures (n = 15).

We generated extrahepatic human cholangiocyte organoids (EHCOs) from the common bile duct remnants of BA patients and controls and grew them as organoids (Figures 1A,B). IF stains confirm that EHCOs are positive for the epithelial marker CK19, and negative for the hepatic marker ALB (Supplementary Figure S1). Patient-derived human organoids clinical data are presented in Supplementary Table S1. Successful propagation rate of BA derived EHCOs was 65%, whereas 91% of the control EHCOs were propagated into viable organoid cultures. In terms of morphology six out of 15 (40%) BA EHCOs demonstrated a deformed organoid shape, compared to one deformed EHCOs out of 11 controls (9%) (Figures 1B,C). EHCOs sizes varied between patients and had a wide range in each culture, ranging from approximately 50–500 microns, regardless of the baseline patients’ characteristics.

To investigate mechanisms of cholangiocyte injury in BA, we used a two-stage design: (i) **discovery**—bulk RNA-seq of extrahepatic cholangiocyte organoids (EHCOs) from 5 BA patients and three controls and (ii) **validation**—replication in an expanded BA/control EHCO cohort together with functional assays. Of note, for immunofluorescent protein analysis we selected morphologically comparable organoids (normal appearing) to minimize growth-related bias.

**FIGURE 2:**
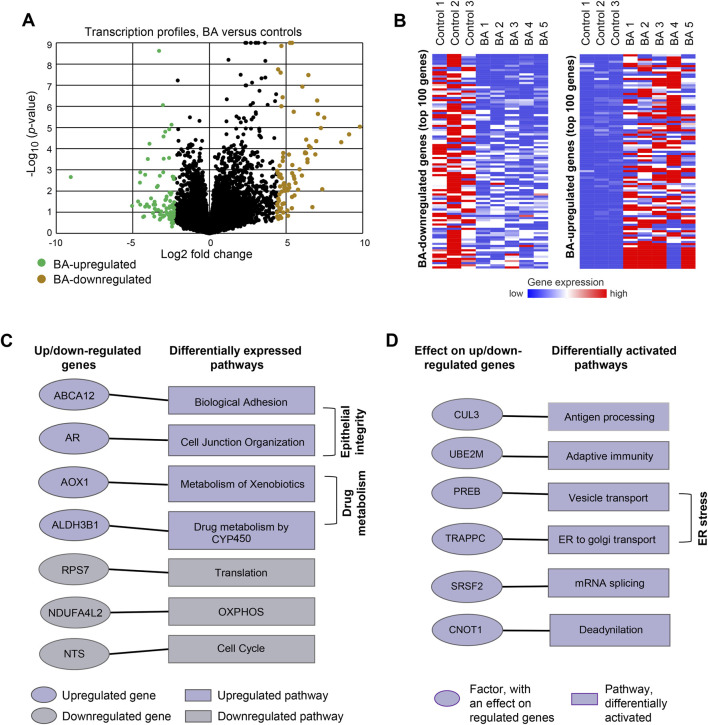
Molecular profiling reveals transcriptomic changes in BA HCOs **(A)** Differential expression between BA compared to controls. Volcano plot showing the comparison of transcription profiles between EHCOs derived from 5 BA patients and three non-BA controls **(B)** Top up- and downregulated genes. A heatmap presenting the expression level (color coded) of each gene (columns) in each individual (rows). Five BA patients and three controls are indicated. Included are top 100 genes that are upregulated (left) or upregulated (right) in BA **(C)** Differentially expressed pathways. Left: examples of up/downregulated genes (color coded; see full list in Supplementary Table S2). Right: examples of pathways enriched with upregulated or downregulated genes (color coded; see full list in Supplementary Table S3). Genes and their associated pathways are connected by edges **(D)** Differentially activated pathways. Left: examples of factors that have an effect on up/downregulated genes in BA (see full list in Supplementary Table S4). Right: examples of pathways enriched with the identified factors. The identified pathways are referred to as “differentially activated pathways” (see full list in Supplementary Table S5). Factors and their associated pathways are connected by edges.

Estimates of the BA-associated change in gene expression (differentially expressed genes) revealed a robust response, including both up- and downregulation of genes (Figure 2A; Supplementary Table S2, Methods. Principal component analysis of the samples are presented in Supplementary Figure S2; Figure 2B highlights the genes with the top fold changes, including the top 100 upregulated and top 100 downregulated genes. Gene set enrichment analysis (elaborated under the Methods section) of the up/downregulated genes showed enriched upregulation of genes in drug-metabolism pathways, such as ‘drug metabolism by cytochrome P450’ (enrichment *q* < 10^–5^), ‘metabolism of xenobiotics by cytochrome P450’ (*q* < 10^–5^), as well as upregulation of pathways related to epithelial integrity, such as ‘biological adhesion’ (*q* < 10^–4^) and ‘cell junction organization’ (*q* < 10^–3^). We also observed enriched downregulation of genes in translation (e.g., ‘ribosome’ *q* < 10^–21^), oxidative phosphorylation (*q* < 10^–6^) and cell cycle (q < 10^–3^) processes (Supplementary Table S3). Next, we applied a differential-activation analysis that integrates the differentially expressed genes in conjugation with an external dataset of transcriptional changes induced by genetic perturbations (**Methods**). This analysis enabled the prediction of factors that are activated or repressed in BA, based on the extent to which perturbation of each factor recapitulates BA-associated transcriptional changes (Supplementary Table S4 **, Methods**). Pathways enriched for these factors, termed ‘differentially activated pathways’, include vesicle transport, such as ‘ER to Golgi transport’ (*q* < 10^–4^, e.g., TRAPPC1), and metabolism of RNA, such as ‘mRNA splicing’ (*q* < 10^–7^, e.g., SRSF2((Figure 2D; Supplementary Table S5).

Overall, based on these predictions, we set to focus on three aspects: epithelial integrity, ER stress, and drug metabolism (Figures 2C,D). We performed further experiments to validate the relevance of these hypotheses. The validation and functional studies were performed with additional EHCOs samples from both BA and control groups (Supplementary Table S1).

### BA EHCOs exhibits aberrant cell polarity and epithelial function

**FIGURE 3:**
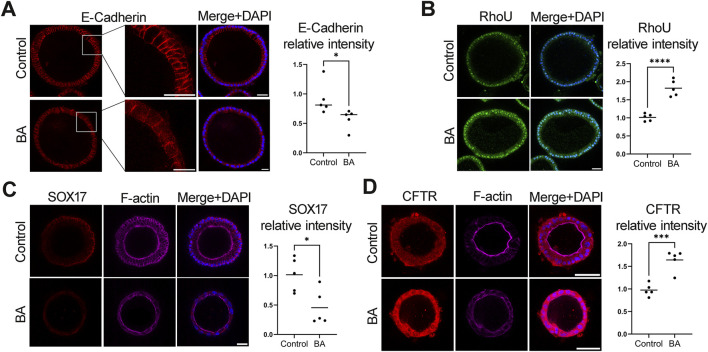
BA-derived EHCOs demonstrate disruption of epithelial polarity. HCOs of BA and controls (n = 5 in each group) were stained for the different polarity markers and quantified based on IF stain. Quantification shows one dot per patient (mean of 10–15 EHCOs quantified by IF intensity per patient) **(A)** IF for E-cadherin (red) and nuclei (DAPI, blue); p = 0.0430 **(B)** IF for RhoU (green) and DAPI (blue); p < 0.0001 **(C)** IF for Sox17 (red), F-actin (magenta), and DAPI (blue); p = 0.0167 **(D)** IF for CFTR (red), F-actin (magenta), and DAPI (blue); p = 0.0005. Scale bar, 50 μm for all panels, except for enlarged E-cad image (panel A) scale bar 100 μm.

Cell-to-cell adhesion and cell polarity are important for the proper epithelial function and regulate tissue homeostasis in critical cell proce3sses that include tissue barrier function, cell proliferation, and migration (Garcia et al., 2018). This is particularly relevant for the biliary tree epithelium, in which improper barrier function may result in bile leak and exposure of cholangiocytes and the sub epithelial layer to bile. E-cadherin is a calcium-dependent adhesion molecule that helps maintain the integrity and polarity of cholangiocytes. It is a key component of adherents junctions and essential to maintain the polarity of the biliary epithelium (Zhou et al., 2021; Merlen and Tordjmann, 2024). EHCOs form BA patients had decreased E-cadherin IF expression compared to normal EHCOs (n = 5, p = 0.0430) (Figure 3A), in addition, Western blot analysis indicated of reduction in E-cadherin protein level in BA EHCOs (Supplementary Figure S3). RhoU, also known as Wrch1, is an atypical member of the Rho GTPase family that plays a crucial role in regulating cell polarity, cytoskeletal dynamics, and cell adhesion) (Hodge and Ridley, 2020; Dickover et al., 2014). RhoU IF stain was increased in BA EHOCs compared to controls (n = 5, p < 0.0001) (Figure 3B). SOX17 is a transcription factor known to be important in cell polarity (Waisbourd-Zinman et al., 2016; Fried et al., 2020; Viotti et al., 2014) EHCOs from BA patients showed decreased expression compared to controls (n = 5, p = 0.0167) (Figure 3C). The cystic fibrosis transmembrane conductance regulator (CFTR) protein is a cAMP regulated chloride and bicarbonate ion channel expressed at the apical plasma membrane of the extrahepatic biliary epithelium, which has a significant role in cell junction formation and actin cytoskeleton organization with its connection to the ECM (Pankonien et al., 2022; Woode et al., 2024). Interestingly, BA EHCOs overexpress CFTR compared to normal controls (n = 5, p = 0.0005) (Figure 3D). This potentially can be a compensatory mechanism to restore epithelial organization as well as improve poor bile flow *via* decreased bile viscosity. The Rhodamine123 assay demonstrates greater luminal contrast leakage in the BA sample than in the control sample (Supplementary Figure S4 and video of the live cell imaging in Supplementary Video S1). Overall, these data demonstrate changes in epithelial integrity and cell junction organization in BA EHCOs compared to controls.

### BA EHCOs demonstrate increased ER stress with abnormal stress response

**FIGURE 4:**
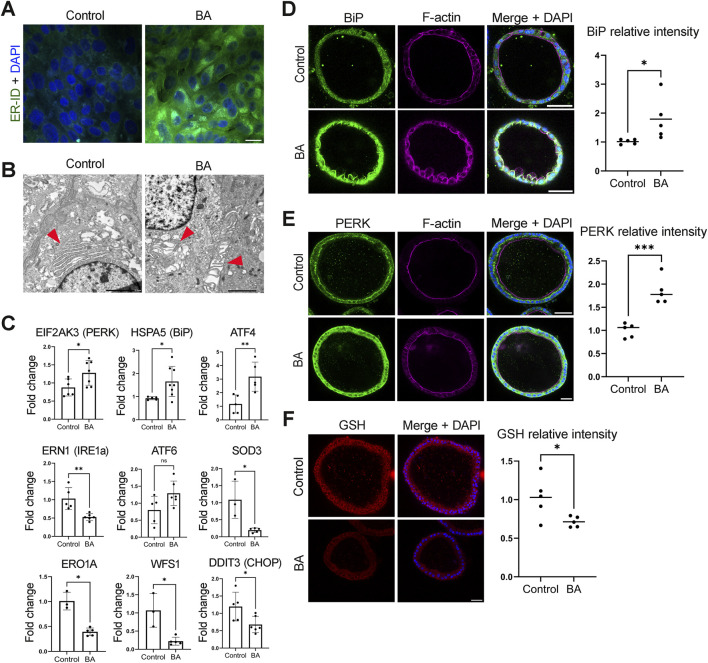
BA-derived EHCOs exhibit increased endoplasmic reticulum (ER) stress **(A)** ER-ID staining of cholangiocytes from BA patients and non-BA controls, n = 4 in each group, scale bar = 20 μm **(B)** TEM images of EHCOs derived from BA patients and non-BA controls. ER is indicated by red arrowheads, n = 3 in each group; scale bar = 2 um **(C)** qRT-PCR analysis of HCOs from BA patients and controls showing expression for EIF2AK3, HSPA5, ATF4, SOD3, ERO1A, WFS1, DDIT3, ERN1 and ATF6. n = 5-8 in BA, 3-6 in non-BA controls, p = 0.0411, p = 0.0295, p = 0.0079, p = 0.0357, p = 0.0357, p = 0.0357, p = 0.0317, p = 0.0043 and p = 0.1255 respectively **(D–F)** Immunofluorescence in EHCOs from BA and non-BA controls; n = 5 patients per group. Quantification shows one dot per patient (mean IF intensity across 10–15 EHCOs per patient). Scale bar, 50 μm **(D)** BiP/HSPA5 (green), F-actin (magenta), DAPI (blue); p = 0.0478 **(E)** PERK/EIF2AK3 (green), F-actin (magenta), DAPI (blue); p = 0.0004 **(F)** Reduced glutathione (GSH, red) and DAPI (blue); at least 40–50 EHCOs counted per condition; p = 0.0355.

Differential regulation analysis revealed changes in the functions of vesicle transport and mRNA splicing in ER of BA EHCOs. The ER is essential for synthesizing, modifying, and folding proteins. Moreover, ER stress can activate the non-canonical WNT signaling pathway leading to disruption of cytoskeleton organization, which plays a role in cell polarity. (Ma et al., 2016). Based on these findings, we investigated ER stress and the associated stress response pathways in BA. We first stained human BA cholangiocytes for the ER IF marker ER-ID, (Batova et al., 2017), which showed higher stain along with condensed ER in BA cholangiocytes compared to controls, suggestive of ER stress (n = 4 in each group) (Figure 4A). We also looked at the ER morphology of EHCOs *via* TEM which revealed dilated and expanded ER folds, also suggestive of ER stress (Ha et al., 2020; Vandewynckel et al., 2015) (n = 3 in each group) (Figure 4B). We then performed qRT-PCR for several ER stress markers. BiP (Binding immunoglobulin protein, also known as GRP78,encoded by HSPA5) is a chaperone protein that can switch from its normal chaperone function to become an ER stress sensor, (Kopp et al., 2019), and found it was upregulated in BA HCOs compared to controls (n = 8 in BA, five in non-BA controls, p = 0.0295) (Figures 4C, 7). Under normal conditions, BiP binds to the luminal domains of unfolded protein response (UPR) sensors (IRE1, PERK, and ATF6), keeping them inactive. During ER stress, BiP dissociates from these sensors to bind misfolded proteins, triggering UPR activation. We thus performed qRT-PCR to those three and found *PERK* (Protein kinase R-like ER kinase, encoded by *EIF2AK3*), and its downstream transcription factor ATF4 (Fusakio et al., 2016) were both upregulated in BA HCOs (n = five to six in BA, five to six in non-BA controls, p = 0.0411, 0.0079 respectively) (Figures 4C, 7). IRE1alpha (Inositol-Requiring Enzyme one alpha, encoded by *ERN1*) is a key transmembrane protein involved in the ER stress response that regulates various cellular processes related to cell survival and death (Junjappa et al., 2018). Interestingly, while it may have been speculated that *ERN1* (IRE1) would increase in a similar manner to *PERK*. *ERN1* gene expression was decreased in BA EHCOs compared to controls (n = 6 in BA, five in non-BA controls, p = 0.0043) (Figures 4C, 7). ATF6 (Activating Transcription Factor 6) plays a pivotal role in linking the ER stress response to oxidative stress, contributing to cellular adaptation and survival under stress conditions (Clark et al., 2020; Haze et al., 1999; McKimpson and Kitsis, 2017; Jin et al., 2017). Thus, it was expected to increase with ER stress, however the change in *ATF6* expression was not significantly different between BA and control samples (n = 6 in BA, five in non-BA controls, p = 0.1255) (Figures 4C, 7). The decrease in *ERN1* (IRE1) and the lack of increase in *ATF6* may occur due to abnormal cholangiocyte response to ER stress or chronic ER stress**.** IF stain for BiP and PERK also showed increased stain in BA EHCOs compared to controls (n = 5 BA, five control, p = 0.0478, p = 0.0004, respectively) (Figures 4D,E). We next examined the downstream effectors of PERK. Wolframin ER Transmembrane Glycoprotein (*WFS1*), *DDIT3* (encodes for C/EBP Homologous Protein (CHOP)) Superoxide Dismutase 3 (*SOD3*) and Endoplasmic Reticulum Oxidoreductase 1 Alpha (*ERO1alpha*), were all decreased in BA EHCOs compared to controls (n = 5 BA, three to five control, p = 0.0357, p = 0.0317, p = 0.0357, p = 0.0357 respectively) (Figure 4C). While these are typically upregulated during prolonged ER stress and function to alleviate cell stress (Hetz, 2012; Han et al., 2013) their decrease may suggest an abnormal ER stress response. In addition, BA derived EHCOs demonstrated lower levels of reduced glutathione (*GSH*) stain compared to non-BA controls (n = 5 BA, five control, p = 0.0355) (Figure 4F). Interestingly, XBP1 expression is not significantly changed in BA EHCOs (log2 fold-change approximately −1.25) with a non-significant adjusted p-value.

### BA HCOs have increased susceptibility to drug/toxic injury which augments stress response

Drug metabolism and cytochrome p450 were noted in the RNAseq analysis to differ between BA and control EHCOs. In order to determine response to drug injury in the EHCOs we used the isoflavonoid toxin biliatresone (Waisbourd-Zinman et al., 2016; Fried et al., 2020; Fried et al., 2025). We previously showed the biliatresone causes lumen obstruction of cholangiocyte spheroids obtained from the extrahepatic cholangiocytes of balb/c mice (Waisbourd-Zinman et al., 2016; Lorent et al., 2015). We were thus intrigued to determine biliatresone effect on EHCOs and to determine if BA EHCOs organoids have increased susceptibility to biliatresone. We counted open EHCOs vs. obstructed ones in culture and compared this ratio pre- and post-biliatresone treatment. Biliatresone resulted in significantly more obstructed EHCOs in BA compared to control EHCOs (n = 3, p = 0.0149, at least 35 EHCOs were counted for each sample) (Figure 6A). We then measured biliatresone-induced ER stress by staining the EHCOs for PERK and E-cadherin. Biliatresone treated BA EHCOs had a higher increase in PERK IF stain (n = 5 BA, 5 control, p = 0.0103 (Figure 6B). E-cadherin IF stain showed an irregular and fragmented pattern (Figure 6B). In order to assess biliatresone effect on ER stress in cholangiocytes, we treated murine neonate extra hepatic primary cell culture with five ug/mL biliatresone, along with one ug/mL thapsigargin, a well known ER stress inducer, and measured ER stress induction by eif2-alpha phosphorylation. Biliatresone induces eif2-alpha phosphorylation after 6 h (Supplementary Figure S5), confirming that biliatresone triggers ER-stress response in cholangiocytes. We then inhibited cytochrome P450 - CYP4A activity *via* the compound HET0016 and measured the previously shown ER stress markers *via* qPCR. *HSPA5*and *EIF2AK3* were decreased with HET0016 treatment (n = 5, p = 0.0079, and p = 0.0079, respectively), while ATF4 and DDIT3 (CHOP) did not significantly altered (p = 0.0635 and p = 0.6825 respectively) (Figure 6C). Lastly, we measured *CYP4A11 via* qPCR and found that BA EHCOs have increased expression compared to controls (n = 5 BA, 3 control, p = 0.0399) (Figure 6D).

### Human BA liver hilum biopsies exhibit increased ER stress proteins

Lastly, though EHCOs were obtained from human extrahepatic bile ducts and it was previously shown that EHCOs maintain their characteristics in culture (Tysoe et al., 2019). We wanted to correlate our findings with human biopsies. We stained liver hilum biopsies from BA patients (removed at the time of KPE) and controls (taken from the liver hilum at the time of liver transplant) and stained for the ER stress markers BiP and PERK. Both were increased in BA samples (n = 3 BA, four control, p = 0.0434, p = 0.0433, respectively) (Figure 7).

## Discussion

In this study, we conducted a comprehensive transcriptomic and functional analysis of extrahepatic cholangiocytes derived from patients with BA and compared them to controls. Our findings revealed key molecular and cellular alterations in the extrahepatic biliary epithelium, highlighting disrupted epithelial integrity, elevated ER stress, and altered drug metabolism as contributors to cholangiocyte susceptibility and injury. Unlike previous studies that focused on intrahepatic cholangiocyte organoids or whole liver transcriptomics, our work specifically examined the extrahepatic biliary tree. This distinction is crucial, as BA manifests primarily as an obstructive disease of the extrahepatic ducts, which have a distinct embryological origin from intrahepatic bile ducts. Additionally, while cholangiocytes can be derived from induced pluripotent stem (iPS) cells, these cells resemble fetal intrahepatic cholangiocytes. In contrast, our organoid model more accurately recapitulates the characteristics of extrahepatic cholangiocytes, as previously demonstrated (Tysoe et al., 2019). This approach provides a unique platform to elucidate the pathophysiological mechanisms underlying BA.

BA-derived EHCOs showed reduced propagation efficiency compared to non-BA controls: 40% out of BA-EHCO cultures formed deformed organoids, and 35% failed to proliferate altogether, consistent with previous findings by Babu et al. (Babu et al., 2020) To minimize growth-related selection bias, we applied identical inclusion criteria to both groups and restricted analyses to passage-matched, morphologically intact, growth-competent EHCOs. This conservative choice strengthens internal validity but may underrepresent severely injured BA epithelia.

The transcriptomic analysis and subsequent validation experiments reveal significant alterations in pathways related to cell junction organization and polarity in BA-derived EHCOs. Specifically, we observed reduced expression of E-cadherin and Sox17, both essential for maintaining cholangiocyte polarity and epithelial integrity, alongside an increase in RhoU, a regulator of cytoskeletal dynamics and adhesion (Garcia et al., 2018; Zhou et al., 2021; Woode et al., 2024). These findings align with our previous work on extrahepatic cholangiocyte injury in a toxic model of biliary atresia, where exposure to biliatresone led to tight junction disruption and increased epithelial permeability, implicating genes in the non-canonical WNT, Notch, and Hippo signaling pathways (Waisbourd-Zinman et al., 2016; Fried et al., 2020; Fried et al., 2025). Supporting this, Amarachintha et al. demonstrated that biliary organoids derived from liver biopsies of BA patients exhibit polarity defects and reduced tight junction integrity in both intrahepatic BA organoids and liver tissues. (Amarachintha et al., 2022). Additionally, genome-wide sequencing analysis by Glessner et al. identified single nucleotide polymorphisms in *AFAP1* and *TUSC3*, key regulators of ciliogenesis and planar polarity, further underscoring the role of disrupted epithelial integrity in BA pathogenesis (Glessner et al., 2023).

Interestingly, CFTR was overexpressed in BA-derived EHCOs. CFTR is a cAMP-regulated chloride (Cl−) and bicarbonate (HCO3−) ion channel expressed at the apical membrane of cholangiocytes. Beyond its ion transport function, CFTR plays a crucial role in establishing and maintaining epithelial apical-basolateral polarity (Pankonien et al., 2022). Roos et al. demonstrated that CFTR activity is suppressed under hypoxic conditions in intrahepatic cholangiocyte organoids (Roos et al., 2021). In our study, CFTR overexpression may represent an adaptive or compensatory response to impaired epithelial architecture or an attempt by extrahepatic cholangiocytes to enhance bicarbonate secretion and reduce bile viscosity. Notably, Amarachintha et al. reported decreased CFTR expression in BA-derived organoids, though these originated from intrahepatic cholangiocytes (Amarachintha et al., 2022). Collectively, these findings suggest that disruptions in cholangiocyte polarity within the extrahepatic biliary tree may increase epithelial permeability and compromise barrier function, potentially exacerbating bile duct injury and accelerating disease progression.

**FIGURE 5:**
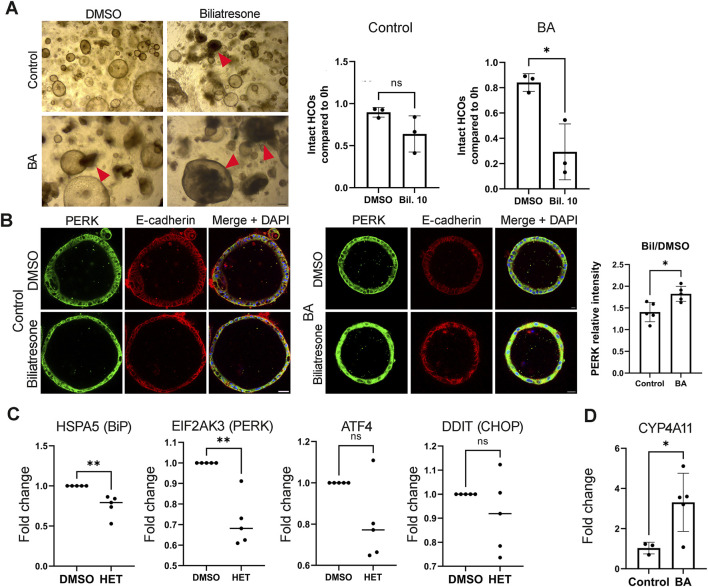
BA EHCOs show heightened drug-induced endoplasmic reticulum stress **(A)** Brightfield images of HCOs from BA and non-BA controls treated with biliatresone, along with quantification EHCOs by morphology (intact lumens vs. obstructed) red arrowheads indicate obstructed lumen, at least 35 EHCOs were counted per condition. n = 3, scale bar = 200 μm **(B)** IF stains for PERK (green), E-cadherin (red), and DAPI (blue) in EHCOs from BA and non-BA controls treated with biliatresone or DMSO, n = 5 for each group, p = 0.0103; scale bar = 50 μm for control and BA upper panel, lower panel = 30 μm. Quantification shows one dot per patient (mean of 10–15 EHCOs quantified by IF intensity per patient) **(C)** qRT-PCR results of ER stress markers in BA EHCOs treated with HET0016. n = 5 in each group, p = 0.0079 (HSPA5), p = 0.0079 (EIF2AK3), p = 0.0635 (ATF4), p = 0.6825 (DDIT3 (CHOP)) **(D)** qRT-PCR expression of CYP4A11 in HCOs from BA and non-BA controls, n = 5 BA; 3 non-BA, p = 0.0399.

A key finding in this study was the evidence of ER stress in BA-derived EHCOs, supported by the upregulation of ER stress markers and morphological changes observed *via* electron microscopy. BiP, a molecular chaperone that alleviates cellular stress by binding misfolded proteins in the ER, (Melo et al., 2022), and PERK, a key sensor of the UPR that reduces protein synthesis under stress conditions (Yi et al., 2017), were both significantly increased in BA-derived EHCOs. Additionally, ATF4, a transcription factor downstream of PERK that regulates restoration of cellular homeostasis after oxidative stress (Wu et al., 2017; Masuda et al., 2013), was also elevated. We thus further looked at the PERK-ATF4 pathway of the UPR (Fusakio et al., 2016). Interestingly, while ATF4 typically induces CHOP expression under stress conditions, CHOP did not increase as expected in BA-derived EHCOs, potentially suggestive of abnormal handling with ER stress. A similar phenomenon has been reported in other contexts: Estébanez et al. demonstrated that exercise increases ATF4 without a corresponding CHOP upregulation in elderly individuals (Estéb et al., 2019). Konsavage et al. showed that hyperoxia increases both ATF4 and CHOP in adult rats but only ATF4 in neonates (Konsavage et al., 2012). While PERK-ATF4 pathway was upregulated, AFT6 and IRE1α were not. AFT6 induces antioxidant genes and lack of ATF6 activation leaves cells more vulnerable to oxidative damage (Jin et al., 2017). The downregulation of IRE1α can attenuate its pro-apoptotic functions (Liu et al., 2023). Both the lack of ATF6 increase combined with IRE1α decrease may result in cell survival rather than apoptosis of cell with increased ER stress. Downstream genes: *ERO1A*, *SOD3*, and *WFS1*, regulators of ER stress and antioxidant defense, were significantly downregulated in BA-derived EHCOs. ERO1A knockout has been shown to impair glutathione redox potential in the ER (Liu et al., 2023). Consistent with this, BA EHCOs exhibited decreased GSH levels compared to controls. We previously showed that decreased GSH leads to upregulation of RhoU/Wrch1,and later downregulation of the transcription factor SOX17 (Fried et al., 2020) (Figure 5). The relationship between ER stress and epithelial polarity has been previously established. ER stress promotes epithelial-mesenchymal transition (EMT) by driving morphological changes, enhancing cell migration, and altering adhesion molecule expression—specifically, downregulating E-cadherin while upregulating N-cadherin and vimentin (Zhou et al., 2020). Furthermore, SOD3 has been shown to preserve adherens junction proteins, including E-cadherin (Tak et al., 2021) (Figure 5). Interestingly, experimental data indicate that IRE1–XBP1 signaling is typically induced early and subsequently attenuated, whereas PERK–ATF4–CHOP can remain active and even predominate during chronic or maladaptive ER stress. In several injury models, XBP1s is only modestly and transiently upregulated, while CHOP and other PERK outputs increase robustly, indicating a PERK-biased response despite ongoing ER stress. Our findings of increased BiP and PERK with no significant change in XBP1 may be consistent with an unbalanced UPR state rather than absence of ER-stress signaling (Fu and Doroudgar, 2022; Chen et al., 2023; Li et al., 2009).

**FIGURE 6:**
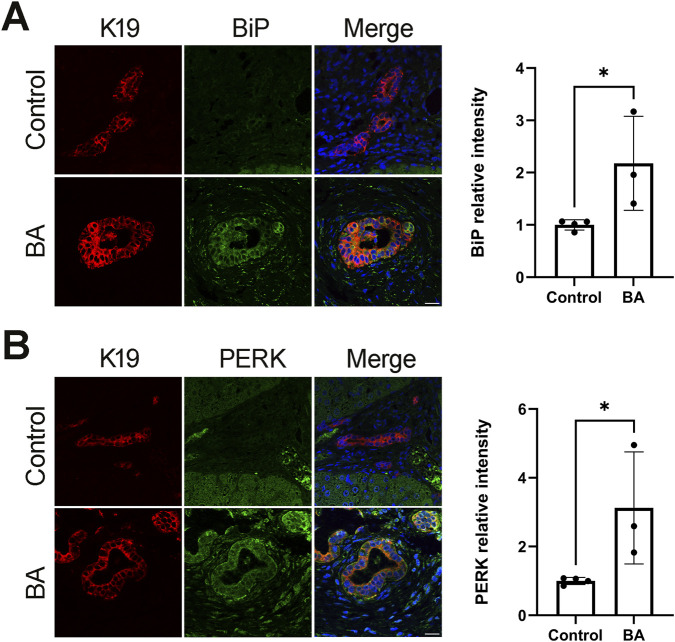
PERK and Bip are overexpressed in liver hilum biopsies from BA patients. Liver hilum biopsies from BA patients (collected at the time of Kasai portoenterostomy) and non-BA controls (collected during liver transplantation) were stained for ER stress markers BiP and PERK **(A)** IF stains of BiP (green), K19 (red), and DAPI (blue), n = 3 for BA, n = 4 for non-BA control, p = 0.434, scale bar = 50 μm **(B)** IF stains of PERK (green), K19 (red), and DAPI (blue). n = 3 for BA, n = 4 for non-BA controls, p = 0.04330, scale bar = 50 μm.

**FIGURE 7:**
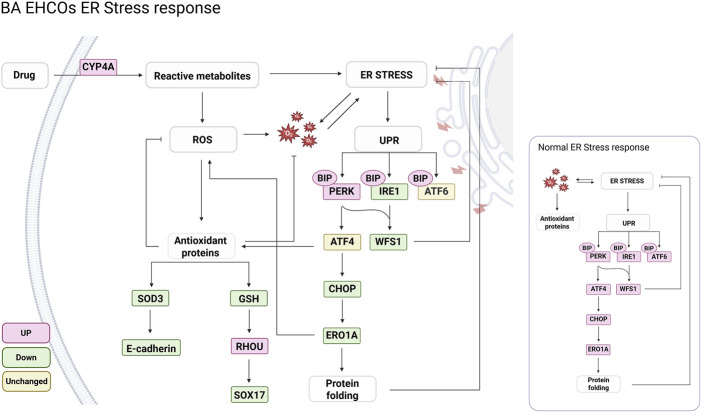
Pathways involved in BA EHCOs mechanism of injury. Summary of genes and pathways that were identified on bulk RNA-seq and further verified between BA and control HCOs. Created with Bio Render.

Altogether, our findings suggest that ER stress is not effectively managed in BA cholangiocytes, and this may result in abnormal cell polarity.

An interesting finding of the RNA-seq analysis comparing BA patients and controls was differences in drug metabolism and xenobiotics pathways. This is of potential interest as BA is thought to result from an environmental factor be it a toxin, drug or virus during the fetal period in predisposed cholangiocytes (Bezerra et al., 2018; Tam et al., 2024). We observed that BA EHCOs demonstrated a more pronounced response to the toxin biliatresone, both by the percentage of morphologically damaged organoids and by the increase in ER stress makers which was higher than that observed in control EHCOs. The ER hosts numerous enzymes, including cytochrome P450 isoforms, that metabolize drugs into more water-soluble forms for excretion. However, in the process of converting xenobiotics, the ER can generate reactive intermediates, free radicals, or other toxic byproducts. These metabolic byproducts can disrupt protein folding, calcium homeostasis, and redox balance within the ER, leading to the accumulation of misfolded or damaged proteins (Huang et al., 2022; Foufelle and Fromenty, 2016). Our study shows that inhibition of CYP4A activity attenuated ER stress markers, suggesting a crosstalk between xenobiotic metabolism and ER homeostasis (Figure 5). A limitation of our work is that protein-level validation beyond E-cadherin remains constrained by the technical challenges of Western blotting in human EHCOs, which provide limited and Matrigel-contaminated protein yields; thus, our conclusions rely primarily on integrated transcriptomic and immunostaining readouts. A potential bias of using organoids as human disease model is the potential misrepresentation of the original tissue phenotype under cell culture conditions. This concern is particularly relevant in the study of BA, which is characterized by patchy obstructions along the common bile duct. Our study demonstrated that while most BA-derived EHCOs appear to have an intact structure on light microscopy similar to control-derived EHCOs, gene expression analyses confirmed that BA-derived EHCOs retain distinctive characteristics long after removal from their original environment. Additionally, staining of liver hilum samples obtained from BA patients during Kasai portoenterostomy and non-BA controls obtained from liver explants highlights the involvement of ER stress in BA. Specifically, ER-related proteins were upregulated in the ductal plates of BA patients compared to non-BA controls. These results reinforce the physiological relevance of EHCOs as a model for studying BA pathogenesis. Prior studies have also demonstrated that organoids faithfully maintain the cellular and molecular features of their tissue of origin, both in cholangiocytes (Tysoe et al., 2019; Liu et al., 2024) and across various other tissues (Chen et al., 2024; Salgueiro et al., 2022).

Taken together, our findings underscore the value of extrahepatic cholangiocyte organoids as a powerful model for studying the pathophysiology of BA. By integrating transcriptomic profiling with functional assays, we reveal key disease-associated alterations, including disruptions in cell polarity, heightened susceptibility to xenobiotic metabolism, and persistent ER stress which is not handled appropriately in BA-derived cholangiocytes. These insights provide a foundation for future research aimed at modulating ER stress responses as a potential strategy to mitigate cholangiocyte injury and improve EHCO function. Importantly, the ability of organoids to retain key pathogenic features of the original tissue highlights their relevance not only for investigating human biliary diseases but also for advancing the development of novel therapeutic interventions.

## Data Availability

RNA sequencing data can be found in the GEO database (accession number GSE276230). Additional results are available from the authors upon request.
