# Supplementary figures and images for: Human biliary atresia extrahepatic cholangiocyte organoids express increased ER and oxidative stress, altered drug metabolism and cell polarity changes
Source: Front Bioeng Biotechnol. 2026 May 7;14:1777423. doi: 10.3389/fbioe.2026.1777423 (PMC13189839; doi:10.3389/fbioe.2026.1777423)

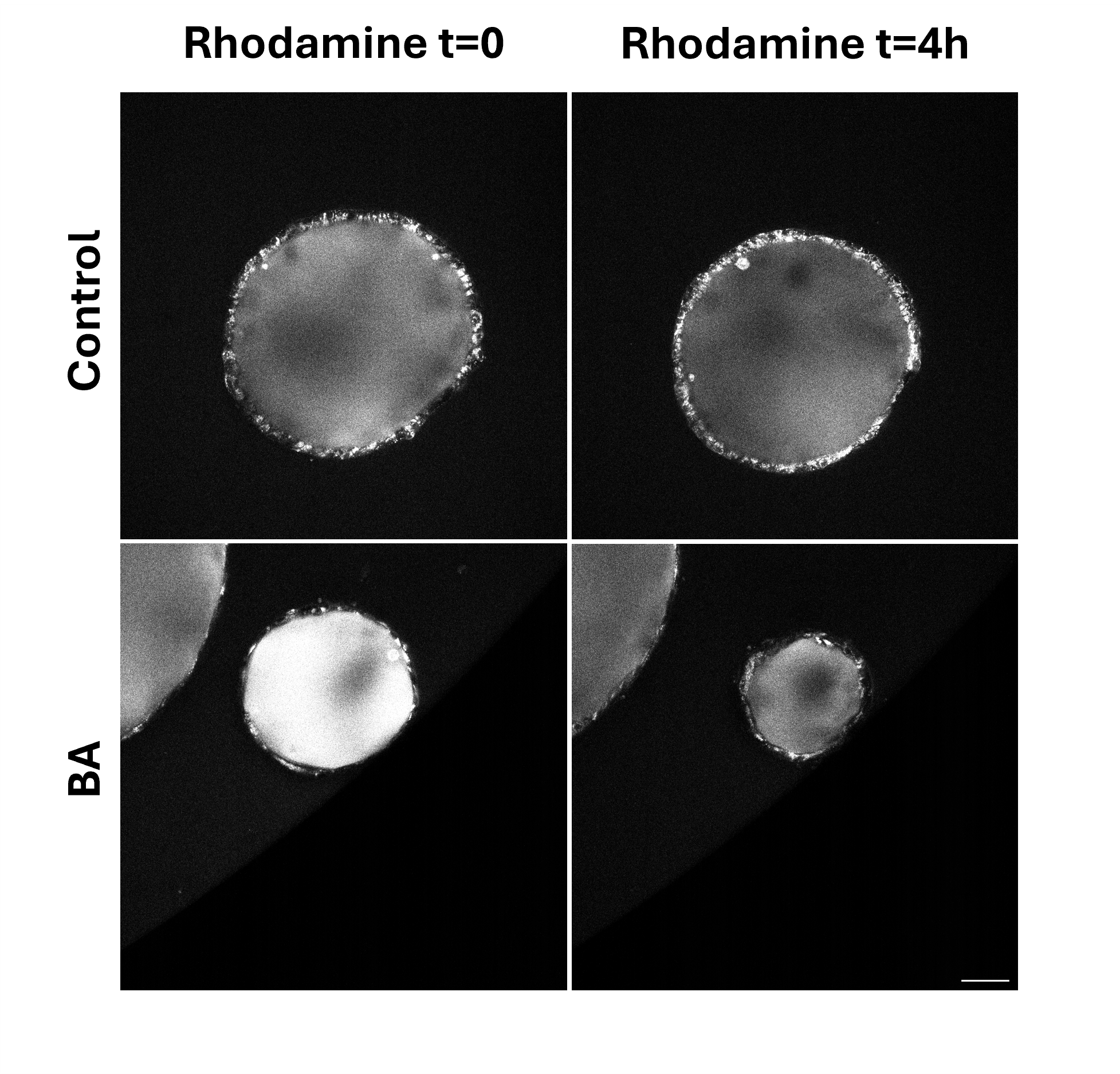

Supplement: Supplementary file 1 [file Image3.tif]

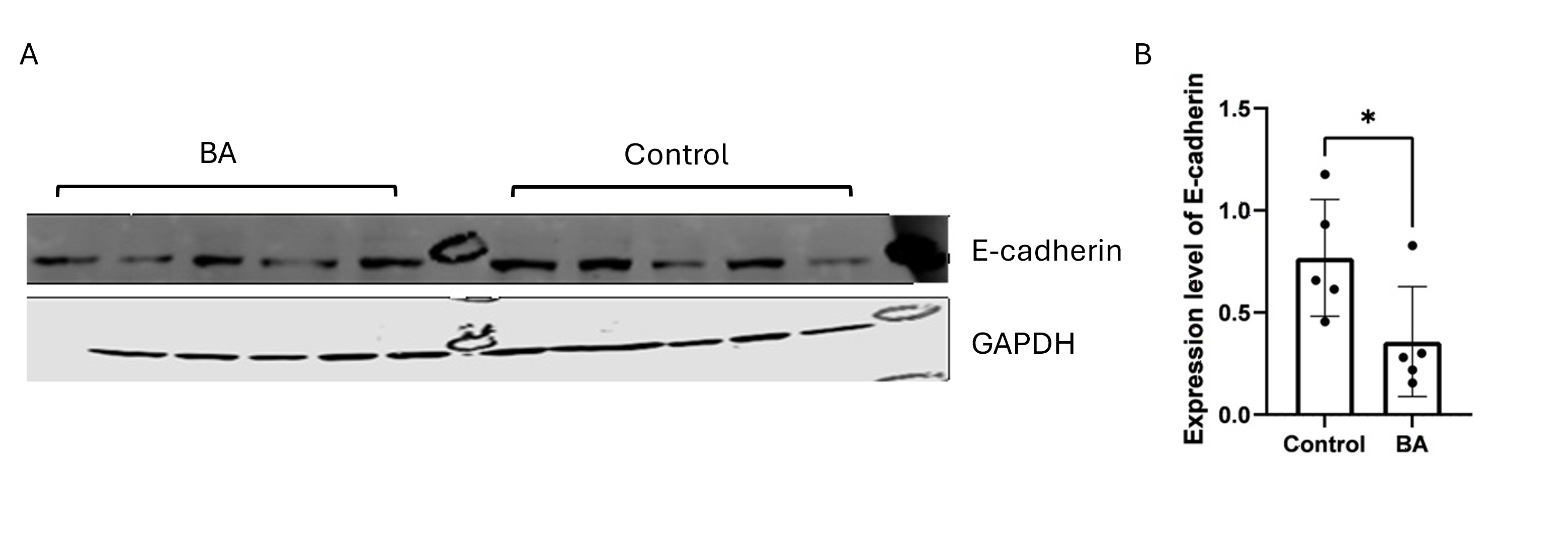

Supplement: Supplementary file 2 [file Image4.tif]

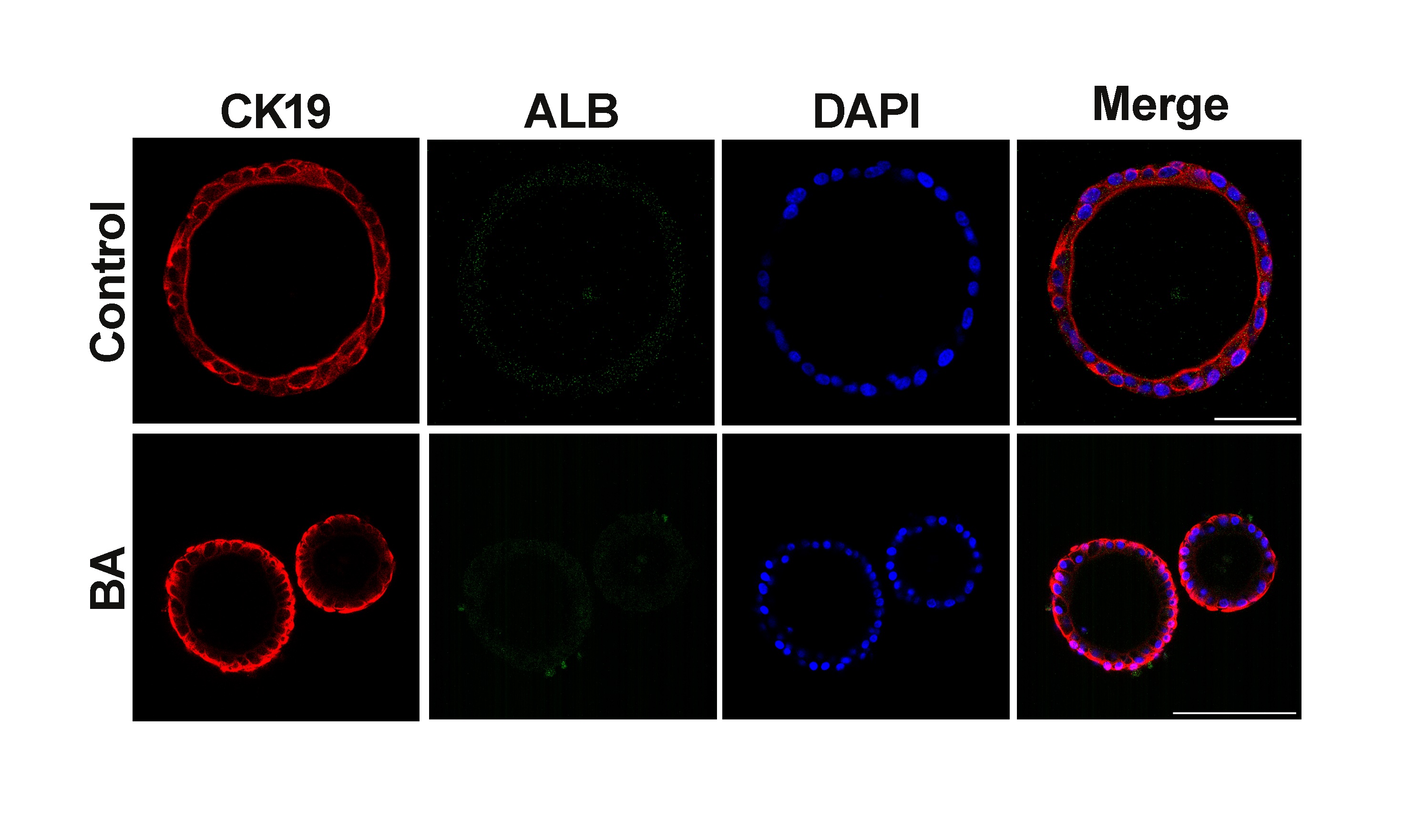

Supplement: Supplementary file 3 [file Image1.jpeg]

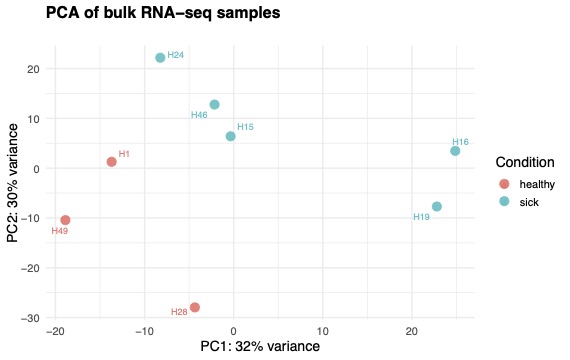

Supplement: Supplementary file 4 [file Image2.jpeg]

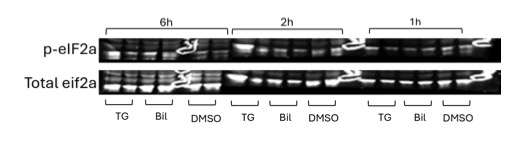

Supplement: Supplementary file 5 [file Image5.jpeg]
