# Supplementary material for: Human biliary atresia extrahepatic cholangiocyte organoids express increased ER and oxidative stress, altered drug metabolism and cell polarity changes
Source: Front Bioeng Biotechnol. 2026 May 7;14:1777423. doi: 10.3389/fbioe.2026.1777423 (PMC13189839; doi:10.3389/fbioe.2026.1777423)
Supplement: Supplementary file 6 [file DataSheet1.pdf]

Supplementary Materials for:

Human biliary atresia extrahepatic cholangiocyte organoids express increased ER and oxidative stress, altered drug metabolism and cell polarity changes

Adi Har-Zahav[1,2,*], Yara Hamoudi[2,*], Keren Danan[3,*], Ana Tobar[4], Michal Bespalchik[2], Michael Gurevich[5], Raanan Shamir[1,2], Irit Gat-Viks[3,¶], Orith Waisbourd-Zinman[1,2,6,¶,#]

1. Gastroenterology, Nutrition and Liver Diseases, Schneider Children's Medical Center, Petach-Tiqva, Israel

2. Felsenstein Medical Research Center, Gray Faculty of Medical and Health Sciences, Tel-Aviv University, Tel-Aviv, Israel

3. The Shmunis School of Biomedicine and Cancer Research, George S. Wise Faculty of Life Sciences, Tel Aviv University, Tel Aviv, Israel.

4. Department of Pathology, Beilinson Medical Center, Petach-Tiqva, Israel

5. Pediatric liver transplant unit, Schneider Children's Medical Center, Petach-Tiqva, Israel

6. Children's Hospital of Philadelphia, University of Pennsylvania, Philadelphia, Pennsylvania, USA

Supplementary Tables

Table S1: Patients' characteristics

ID	Age	Sex	Clinical data	Tissue source
H1	3y	Female	Budd-Chiari syndrome	Liver transplantation
H15	7w	Male	Biliary atresia	KPE
H16	8w	Male	Biliary atresia	KPE
H19	7w	Male	Biliary atresia	KPE
H21	16y	Male	Wilson	Liver transplantation
H24	8w	Male	Biliary atresia	KPE
H28	4y	Female	ARPKD	Liver transplantation
H44	1y	Male	MSUD	Liver transplantation
H46	7w	Male	Biliary atresia	KPE
H48	6w	Female	Biliary atresia	KPE
H49	8y	Female	Autoimmune Hepatitis	Liver transplantation
H51	9w	Male	Biliary atresia	KPE
H53	7w	Female	Biliary atresia	KPE
H54	4y	Female	Autosomal recessive polycystic kidney disease	Liver transplantation
H56	4Y	Male	Langerhans cell histiocytosis	Liver transplantation
H73	6.5w	Female	Biliary atresia	KPE
H77	6w	Female	Biliary atresia	KPE
H82	1.5y	Female	Hyperoxaluria	Liver transplantation
H83	8w	Female	Biliary atresia	KPE
H84	1.5y	Male	Hepatoblastoma	Liver transplantation
H89	3y	Female	Hepatoblastoma	Liver transplantation

Table S6: Supplemented Williams' E Media

	Final concentration	Catalig number	Supplier	Comments
Nicotinamide	10 mM	N0636-100G	Sigma-Aldrich	
Sodium bicarbonate	17 mM	S6014-500G	Sigma-Aldrich	
Ascorbic acid tri-sodium 100mM salt stock solution	0.2 mM	49752-10G	Sigma-Aldrich	
D- Glucose	14 mM	15023021	Gibco	
Sodium pyruvate	6.3 mM	11360070	Gibco	
HEPES solution	20 mM	15630056	Gibco	
ITS+ premix	1:100	354352	Corning	
Dexamethasone	0.1 uM	1126/100	R&D Systems	
Glutamax	2 mM	35050061	Gibco	
Pen-strep	100 U/ml - 100 ug/ml	15140122	Gibco	
R-Spondin1	500 ng/ml	120-38	PeproTech	Freshly added
DKK1	100 ng/ml	120-30	PeproTech	Freshly added
EGF	20 ng/ml	AF-100-15	PeproTech	Freshly added
Y-27632 dihydrochloride	10 uM	HY-10583	MCE	Freshly added

Table S7: List of qPCR primers

Gene	Forward Primer	Reverse Primer
HPRT	TATGGCGACCCGCAGCCCT	CATCTCGAGCAAGACGTTCAG
ERO1A	GCCAGGTTAGTGGTTACTTGG	GGCCTCTTCAGGTTTACCTTGT
SOD3	ATGCTGGCGCTACTGTGTTC	CTCCGCCGAGTCAGAGTTG
WFS1	GCGACACGGATGAAGAACTC	CTCACCTCCCGTTCGTTCT
H5PA5 (BiP)	TGTTCAACCAATTATCAGCAAACTC	TTCTGCTGTATCCTCTTCACCAGT
EI2AK3 (PERK)	ATGAGACAGAGTTGCGACCG	TGGATGACACCAAGGAACCG
ATF4	GTTCTCCAGCGACAAGGCTA	ATCCTGCTTGCTGTTGTTGG
ATF6	CAAATAGCCAACAGAAAGCCC	TAATACACTTGCAGCTCACTCCC
ERN1 (IRE1a)	CCTACAAGAGTATGTGGAGC	GGTCTCTGTGAACAATGTTGAGAG
DDIT (CHOP)	AGAACCAGGAAACGGAAACAGA	TCTCCTTCATGCGCTGCTTT
CYP4A11	ACGGCTTGCTCCTGTTGAATGG	AGAGGTCAGGCTGTAGATGGTGTC

Table S8: List of antibodies

Antibody	Species	Company / Product #	Dilution
PERK	Rabbit	Proteintech / 24390-1-AP	1:200
RhoU	Rabbit	Abcam / ab80315	1:150
BIP	Rabbit	Proteintech / 11587-1-AP	1:200
E-Cadherin	Mouse	Abcam / ab231303	1:200
GSH	Mouse	Abcam / ab19534	1:150
SOX17	Mouse	Abcam / ab192453	1:150
CFTR	Mouse	Abcam / ab2784	1:200
KRT19	Mouse	Abcam / ab7754	1:200
Alexa Fluor 488	Goat Anti-Rabbit	Abcam / ab150081	1:500
Alexa Fluor 555	Gout Anti-Mouse	Abcam / ab150118	1:500
Alexa Fluor 647	Goat Anti-Mouse	Abcam / ab150119	1:500
DAPI	Nuclear stain	Invitrogen / D1306	1:1000
phalloidin tetramethyl rhodamine B isothiocyanate		Santa Cruz Biotechnology / #301530	Final conc. 50 ng/ml

Supplementary Methods

RNA-seq data of BA patients

The dataset consists of bulk RNA-seq profiles from EHCOs originating from patients with BA undergoing Kasai portoenterostomy and normal ducts from patients undergoing liver transplantation for metabolic conditions. For all samples, we used FASTQ files of the forward DNA strand (length 67bp). Quality control was performed using FastQC. As evidence for the quality of data, we observed the following metrics: on average, each sample contains 30.7M reads, with no reads flagged as poor quality, and the average GC content across samples was 51%.

Alignment of reads to the reference genome (hg38.refGene) was conducted using the STAR aligner, resulting in a reads-per-gene count matrix. Both the raw sequencing data and the count matrix were deposited in GEO (accession number GSE276230). The average mapping ratio was 83.54%, with a total of 28,271 genes detected. Gene filtration and normalization were performed using the DESeq2 R package, with default filtering settings and a minimum read count threshold of 10 reads per gene. After filtration, 16,896 genes were retained for subsequent analysis.

Analysis of differential expression in BA

Differential expression (DE) analysis of BA versus controls was performed through DeSeq2 in R (**Table S2**). For the log2FC value, positive/negative sign indicates up-regulation or down-regulation in BA compared to healthy samples. For each pathway (from KEGG and the GO repositories), we calculated the bias of the log2FC scores for genes within the pathway compared with the remaining genes. The bias is quantified using the Wilcoxon rank-sum test (FDR-adjusted *q* values). The 'differentially expressed pathways' are pathways with *q*-value < 0.005. Enriched

pathways are further classified as either upregulated or downregulated pathways according to the direction of bias in BA – namely, 'upregulated/downregulated pathways' in BA are those that are enriched with genes that have high/low log2FC scores (**Table S3**).

Pre-processing of reference data

We used perturb-seq data, which targets genes with CRISPR interference (CRISPRi) with subsequent transcription phenotyping at single cell resolution.[1] The experiment was performed in the retinal pigment epithelial (RPE1) cell line. It is one of the largest single-cell CRISPRi Perturb-seq resources available, with systematic transcriptional consequences of thousands of perturbations in a polarized epithelial context. We treated the RPE1 Perturb-seq resource as a cross-epithelial reference because many core epithelial programs behave similarly across tissues. We identified the top up- and down-regulated genes in BA EHCOs (detailed below) and assessed their concordance with gene-expression changes induced by specific CRISPRi knockdowns

We used as input the processed data after various filtration and pre-processing steps as reported in Replogle *et al.,* 2022. The input data, which is used as input in our study, consists of a z-score for each gene in each single cell. The z-scores were obtained by normalization to control cells. The input data include 2393 perturbations, each of which was measured in 2 to 3461 single cells; each single cell is represented by a vector of z scores across 8748 transcribed genes. In addition, the input data includes non-targeting control cells.

For each genetic perturbation *r*, we applied the following two steps. First, we calculated the effect of the perturbation in gene *r* on each transcribed gene *g* by comparing the z scores of gene g in all single cells in which *r* is perturbed against all non-targeting (control) single cells (p-value of Wilcoxon rank-sum test, q-value after FDR correction for multiple genes). The 'perturbation effect score' of a perturbation *r* on a gene *g* is defined as the signed log10 of this *q*-value, with

positive/negative perturbation effect scores for increase/decrease in the median values of gene *g* in *r*-perturbed cells compared to non-targeting cells. Each perturbation *r* is represented with a 'perturbation profile', consisting of a 8748-length list of the perturbation effect scores for perturbation *r* across all 8748 transcribed genes. A total of 2393 perturbation profiles were calculated, a profile for each perturbed gene. These profiles were used as input in the analysis of differential activation in BA.

Analysis of differential activation in BA

The analysis takes as input: (i) A BA gene sets: either a BA-upregulation gene set, including the 100 genes with highest log2FC scores (denoted BA-Up), or the BA-downregulation gene set, including the 100 genes with lowest log2FC scores (denoted BA-Down) (**Table S2**). (ii) Perturbation effect scores of a given factor. This reference data consists of the perturbation profiles of a factor across the 8748 transcribed genes. Of note, only 18 of the 100 BA-Up genes and only 10 of the 100 BA-Down genes were expressed in perturbed single cells and were therefore used in the subsequent calculation.

Given an input BA gene set (BA-Up or BA-Down sets) and a perturbation profile of a given factor, the 'effect of perturbation on a BA gene set' is defined as the bias of the perturbation effect scores of the perturbed factor on genes within the BA gene set compared to the remaining genes (a Wilcoxon rank-sum test *p* value) (**Table S4**). Thus, a perturbation upregulates/downregulates the BA gene set when the genes in the BA gene set tend to high/low perturbation effect scores. We distinguish four types of factors with a significant effect (**Table S4**): Factors whose perturbation (1) downregulates the BA-Down's genes (25 factors, effect on BA-Down p <0.11); (2) downregulates the BA-Up's genes (48 factors, on BA-Up $p<0.01$); (3) upregulates the BA-Down's genes (25 factors, effect on BA-Down $p< 0.07$); and (4) upregulates the BA-Up's genes (2 factors, effect on BA-Up $p<0.08$). For the factors within each of these categories, we performed hyper-

geometric test using each of the pathways of the REACTOME collections (**Table S5**). Pathways that are enriched (q <0.05) are referred to as 'differentially activated pathways'.

1. Replogle JM, Saunders RA, Pogson AN, et al. Mapping information-rich genotype-phenotype landscapes with genome-scale Perturb-seq. *Cell*. 2022;185(14):2559-2575.e28.
